# Correlation of SARS-CoV-2 Viral Load and Clinical Evolution of Pediatric Patients in a General Hospital From Buenos Aires, Argentina

**DOI:** 10.3389/fped.2022.883395

**Published:** 2022-07-07

**Authors:** Martín Eduardo Brizuela, Sandra Elizabeth Goñi, Georgina Alexandra Cardama, María Alejandra Zinni, Alejandro Andres Castello, Leandro Matías Sommese, Hernán Gabriel Farina

**Affiliations:** ^1^Servicio de Infectología, Hospital Zonal General de Agudos “Dr. Isidoro G. Iriarte”, Quilmes, Argentina; ^2^Laboratorio de Virus Emergentes, Departamento de Ciencia y Tecnología, Universidad Nacional de Quilmes, Bernal, Argentina; ^3^Plataforma de Servicios Biotecnológicos, Departamento de Ciencia y Tecnología, Universidad Nacional de Quilmes, Bernal, Argentina; ^4^Departamento de Ciencia y Tecnología, Universidad Nacional de Quilmes, Bernal, Argentina; ^5^Grupo de Bioinformática Estructural, Universidad Nacional de Quilmes, Bernal, Argentina

**Keywords:** COVID-19, SARS-CoV-2, children, viral load, cycle threshold (CT) value

## Abstract

**Background:**

SARS-CoV-2 infection is associated with a wide range of clinical manifestations and severity. Pediatric cases represent <10% of total cases, with a mortality rate below 1%. Data of correlation between SARS-CoV-2 viral load in respiratory samples and severity of disease in pediatric patients is scarce. The cycle threshold (CT) value for the detection of SARS-CoV-2 could be used as an indirect indicator of viral load in analyzed respiratory samples.

**Objective:**

The aim of this study was to describe CT values and their correlation with clinical manifestations, epidemiology and laboratory parameters in pediatric patients with confirmed COVID-19.

**Methods:**

In this observational, retrospective, analytic and single-center study we included patients under 15 years with confirmed COVID-19 by RT-PCR SARS-CoV-2 admitted to the Isidoro Iriarte Hospital (Argentina) between March 1st 2020 and April 30th 2021.

**Results:**

485 patients were included, the distribution according to disease severity was: 84% (408 patients) presented mild disease, 12% (59 patients) moderate disease and 4% (18 patients) severe disease. Patients with moderate and severe illness had an increased hospitalization rate, prolonged hospitalization, higher frequency of comorbidities and oxygen and antibiotics use. CT values, that could be used as an indirect measure of viral load, was associated with severity of clinical manifestations and age under 12 months. No patient required admission to PICU nor mechanical ventilation. No deaths were registered.

**Conclusions:**

In this study, the viral load of SARS-CoV-2 in respiratory samples, determined by the cycle threshold, was significantly correlated with moderate to severe cases and with age.

## Introduction

Cases of COVID-19 caused by severe acute respiratory syndrome coronavirus 2 (SARS-CoV-2) were first reported in late-December 2019. In March 2020 the World Health Organization (WHO) declared the COVID-19 outbreak as a pandemic. By August 12th 2021, 200 million cases and more than 4 million deaths were reported worldwide and 5 million cases and more than 100 thousand deaths in Argentina (1). In Argentina, 10% of the cases affecting people under 19 years of age with a fatality rate of 0.05% mainly associated with comorbidities and age (age group under 12 months) (2).

In the pediatric population COVID-19 severity was associated with the presence of certain comorbidities such as cancer, diabetes mellitus, neurological diseases, among others (3, 4).

The CT (cycle threshold) values in a RT-PCR (reverse transcription real time polymerase chain reaction) assay is defined as the number of cycles required for the fluorescent signal to cross a certain threshold. CT levels are inversely proportional to the amount of target nucleic acid in the sample and can be an indirect measure of viral load of the samples. Although there is no direct viral load quantification, it can be extrapolated that each 3.3 increase in CT value correlates with ~1 log (i.e., 10-fold) less target in the nasopharyngeal sample undergoing the PCR. In line with this idea, it is possible to determine CT ranges of the different samples as indicators of high (CT below <25), moderate (CT between 25 and 35) and low (CT over 35) viral loads.

Since COVID-19 outbreak, several reports have tried to address whether viral load and illness severity are correlated in adult and pediatric patients. The aim of this study is to contribute with this topic focusing on the correlation of SARS-CoV-2 viral load in respiratory samples with clinical severity. Additionally, epidemiological, clinical characteristics and laboratory parameters of COVID-19 positive patients are analyzed.

## Methods

### Study Design

An observational, retrospective and analytic study was conducted including patients aged <15 years with a RT-PCR SARS-CoV-2 test positive who attended to the Hospital Zonal General de Agudos “Dr. Isidoro G Iriarte” from Quilmes, Buenos Aires, Argentina between March 1st, 2021 and April 30th, 2021.

#### Inclusion Criteria

Patients with confirmed SARS-CoV-2 infection by RT-PCR in samples from the upper respiratory tract.

#### Exclusion Criteria

Clinical or epidemiological COVID-19 diagnosis, diagnosis by immunochromatographic methods or patients with incomplete clinical data.

#### Definition

Severity of COVID-19 was defined by clinical and complementary evaluation according to Dong et al. (5).

(1) Asymptomatic infection: no symptoms nor clinical signs, normal chest X-ray, positive SARS-CoV-2 RT-PCR; (2) mild: acute infection symptoms in the upper respiratory tract, including fever, fatigue, myalgia, cough, odynophagia, rhinorrhea, normal auscultation. Some cases presented no fever or had only digestive symptoms such as abdominal pain, diarrhea, nausea, and vomiting; (3) moderate: Pneumonia, persistent fever and cough-mainly dry cough followed by productive cough-, wheezing without dyspnea nor hypoxemia. Asymptomatic cases with subclinical pulmonary lesions determined by chest CT scan; (4) severe: early respiratory symptoms, fever and cough, presented with gastrointestinal symptoms such as diarrhea. Disease progression observed within 1 week reaching dyspnea with central cyanosis. Oxygen saturation of ≤92% with other hypoxia manifestations; (5) or critical: rapid progression to acute respiratory distress syndrome or respiratory insufficiency, shock, encephalopathy, myocardial lesion or cardiac insufficiency, coagulation disorders and acute renal lesions.

Detection of SARS-CoV-2 RNA from samples from the upper respiratory tract was performed by RT-PCR. Nasopharyngeal swabs (combined swab) or nasopharyngeal aspirates (patients under 1 year) were collected following institutional and national recommendations. Samples were analyzed in the COVID-19 Unit of the Universidad Nacional de Quilmes (UNQ). Different commercial kits were used: GeneFinder™ COVID-19 Plus RealAmp kit (OSANG Healthcare, Korea) and Quantabio Script One-Step RT-PCR (Genbiotech SRL) for RNasa P detection; Real-Time Fluorescent RT-PCR Kit for Detecting SARS-CoV-2 (BGI, China); DisCoVery SARS-CoV-2 RT-PCR detection kit Rox o Cy5 (AP-Biotech, Argentina); COVID-19 One-Step RT-PCR Kit FAPON (FAPON Biotech Inc, China). Viral load on respiratory samples was estimated using cycle threshold (CT): high viral load (CT < 25), intermediate viral load (CT 25–35) and low viral load (CT > 35). The samples were analyzed according recommendations of manufacturer.

Different kits were used to detect SARS-CoV-2 RNA due availability of resources in the laboratory and the province of Buenos Aires. All of these commercially available tests were approved by the Ministry of Health of the Province of Buenos Aires and the Administración Nacional de Medicamentos, Alimentos y Tecnología Sanitaria (ANMAT). All of these kits are proven to have high efficiency. Given the differences found in cut-off values among the different commercial kits, in this work we analyzed ranges of CT values to circumvent this issue. Three different CT ranges were used <25, 25–35, and >35, to address high, moderate or low viral loads.

### Statistical Analysis

Continuous variables were analyzed using the *t*-test or the Wilcoxon test for those with parametric normally distributed or non-parametric distributed data, and were expressed as a mean and standard deviation or median and interquartile range, respectively. For categorical variables Chi-square test with Yates or Fisher correction was used and these were expressed as percentages. Univariate analysis was performed to identify potential predictors of severity and a multiple logistic regression model was performed to identify predictive variables for severe infection and COVID-19 fatality in the pediatric population analyzed. Variables with significant association in the initial analysis (*p* < 0.2) and/or those considered to be clinically relevant were analyzed using Wald test. To this realized model, we sequentially added age and comorbidities and other clinical or analytical parameters that were significant in the univariate analysis. All the variables were analyzed individually to find an independent relation with outcomes. A value of *p* < 0.05 was considered significant.

### Ethics

This study was approved for the Research Committee Board of the Hospital Isidoro Iriarte Number 65/2020. The information was processed following the guidelines of the Helsinki Declaration, the CIOMS guidelines and the national laws that determine the handling of people's data. The signing of an informed consent was not required as it was considered a low-risk investigation, with no interventions outside of standard care.

## Results

**Figure 1:**
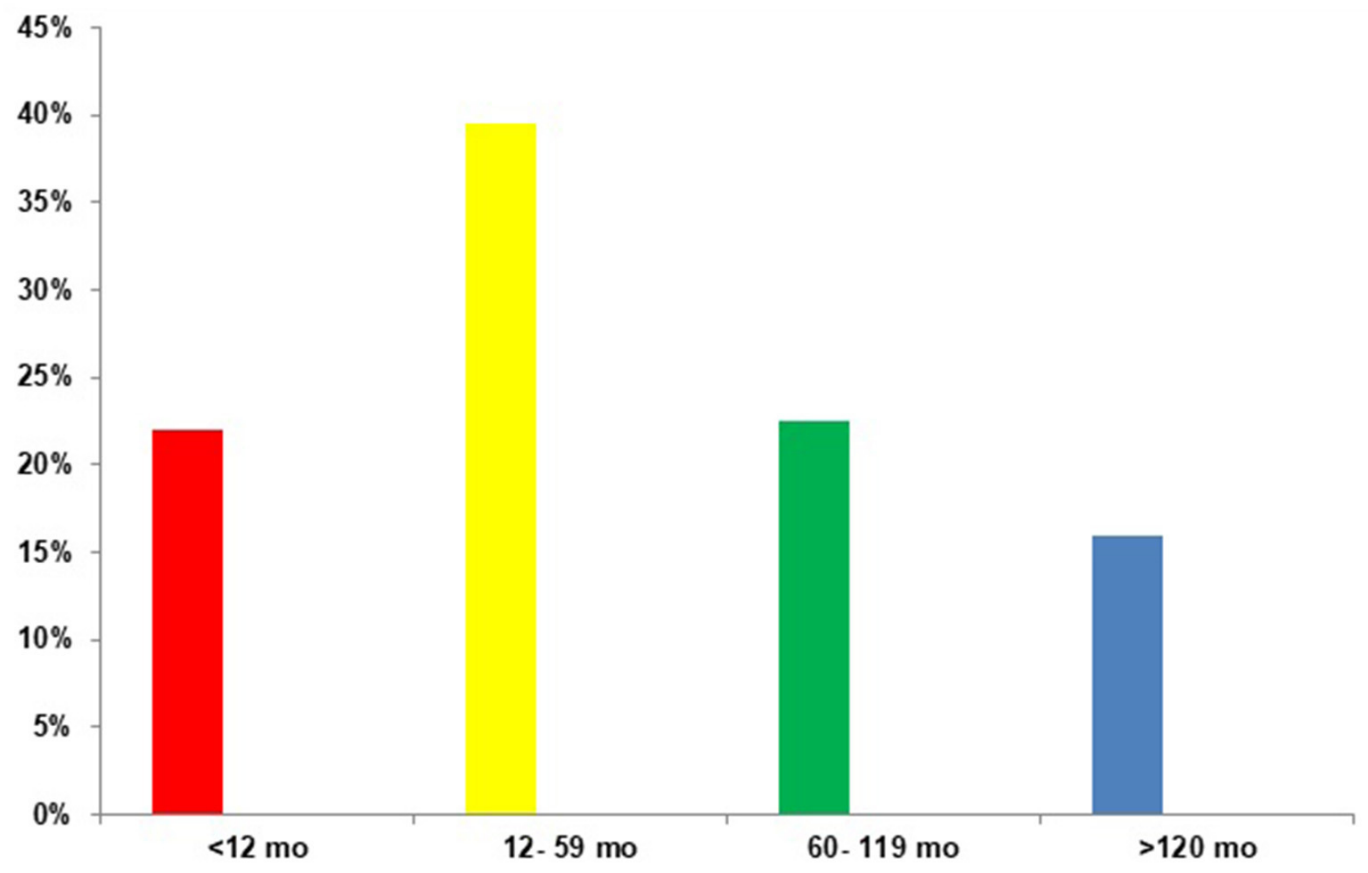
Distribution of the included population according to age (*n* = 485).

The study population included 485 patients (p), 53% (257 p) were male infants with a median age of 36 months. The distribution according to age is shown in Figure 1. All cases were community transmitted, identifying household/family transmission in 23% of them (110 p). All patients were symptomatic, being the most common symptoms at the onset of illness fever [398 p, (82%)], cough [252 p, (52%)], vomiting [145 p, (30%)], diarrhea [116 p, (24%)], dyspnea [87 p, (18%)], odynophagia [82 p, (17%)], abdominal pain [78 p, (16%)] and headache [63 p, (13%)]. According to the clinical severity, 84% (408 p) of the cases were mild, 12% (59 p) moderate and 4% (18 p) severe. No critical patients were observed. Respiratory symptoms were associated with moderate and severe cases (*p* < 0.01), while gastrointestinal symptoms were more frequent in mild cases (*p* < 0.001). Interestingly, 21% (103 p) of patients had underlying comorbidities and these patients presented moderate and severe cases (*p* < 0.001).

Of the 485 analyzed patients, 20% (97 p) required hospital admission and patients with moderate and severe illness had more frequent and prolonged hospitalization (*p* < 0.05). No significant differences were observed in hospital admission rates for all age groups. From hospitalized patients, 10% (49 p) required oxygen therapy and 6% (28 p) received antimicrobial therapy, being more frequent in severe cases (*p* < 0.05).

Interestingly, patients with moderate and severe illness showed a higher viral load (*p* < 0.001). In this regard, the viral load was higher in those under 12 months of age.

**Figure 2:**
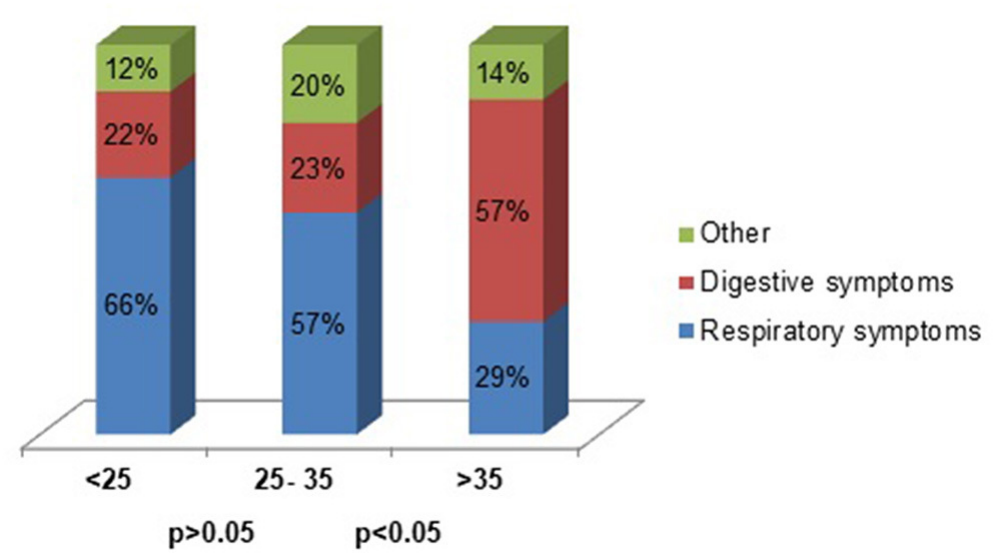
CT PCR-SARS-CoV-2 and predominant symptoms (*n* = 485).

No patients required to be transferred to the pediatric intensive care unit (PICU) and no patient died. The variables associated with clinical and age stratification are shown in Tables 1, 2, respectively, and the distribution of symptoms according viral load (CT-PCR-SARS-CoV-2) is shown in Figure 2.

**Table 1:** Presenting characteristics of the analyzed population.

**Variable**	**Mild** **(*n* = 408)**	**Moderate** **(*n* = 59)**	**Severe** **(*n* = 18)**	***p***
Males (*n*, %)	212 (52 %)	37 (63%)	8 (44%)	NS
Age in months (median, IQR)	36 (17–108)	36 (14–108)	42 (16–87)	NS
Comorbidities (*n*, %)	65 (16%)	31 (53%)	7 (39%)	<0.001
Days of evolution (median, IQR)	4 (3–5)	1(1–2)	2 (1–3)	NS
Hospitalization (*n*, %)	23 (5%)	56 (95%)	18 (100%)	<0.05
Hospitalization days (median, IQR)	3 (2.3–3.7)	4 (3–5)	5 (3–7)	<0.05
Supplementary oxygenation (*n*, %)	5 (1%)	29 (49%)	15 (83%)	<0.001
Days of supplementary oxygenation (median, IQR)	2 (0.9–3.1)	2 (1.7–2.3)	2 (1.56–2.44)	NS
Antibiotic treatment (*n*, %)	5 (1%)	12 (20%)	11 (61%)	<0.05
Days of antibiotic treatment (median, IQR)	5 (1.3–8.7)	5 (2.7–7.3)	5 (3.5–6.5)	NS
Cycle threshold (*n*, %)				<0.001
<25 25–35 >35	58 (14%) 278 (68%) 72 (18%)	19 (32%) 35 (59%) 5 (9%)	12 (67%) 6 (33%) 0 (0%)	
Fever (*n*, %)	357 (87.5)	33 (57)	10 (56)	<0.001
Cough (*n*, %)	198 (48.5)	40 (66)	14 (78)	<0.01
Odynophagia (*n*, %)	79 (19.4)	4 (7)	0 (0)	<0.05
Respiratory distress (*n*, %)	48 (11.8)	27 (45.8)	13 (72)	<0.001
Diarrhea (*n*, %)	111 (27.7)	3 (5)	1 (5.6)	<0.001
Vomiting (*n*, %)	140 (35)	6 (10)	1 (5.6)	<0.001

*Distribution according to disease severity (n = 485)*.

**Table 2:** Clinical and laboratory characteristics distributed by age group of patients (*n* = 485).

**Variable**	** <1 months** **(*n* = 106)**	**13–60 months** **(*n* = 191)**	**61–120 months** **(*n* = 108)**	**>120 months** **(*n* = 80)**	***P***
Males (*n*, %)	60 (57)	104 (54.4)	59 (54.6)	36 (45)	NS
Comorbidities (*n*, %)	13 (12)	46 (24)	27 (25)	16 (20)	NS
Evolution days (median, IQR)	1 (1–2)	1 (1–3)	1 (1–2.25)	2 (1–3)	NS
Hospitalization (*n*, %)	22 (20.8)	38 (20)	27 (25)	10 (12.5)	NS
Days of hospitalization (median, IQR)	4 (3-5)	4 (2.5–4)	3 (2.5–5)	5 (3.25–5.75)	NS
Supplementary oxygen (*n*, %)	8 (7.5)	22 (11.6)	14 (13)	5 (6.25)	NS
Antibiotic therapy (*n*, %)	9 (8.5)	7 (3.7)	7 (6.5)	5 (6.25)	NS
Days of antibiotic therapy (median, IQR)	4 (4–7)	7 (6–7)	5 (5–7)	7 (5–7)	NS
CT (*n*, %)					
<25 (high viral load)	26 (25%)	47 (25%)	23 (21%)	7 (9%)	<0.05
25–35 (intermediate viral load)	68 (64%)	125 (65%)	70 (65%)	56 (70%)	
>35 (low viral load)	12 (11%)	19 (10%)	15 (14%)	17 (21%)	
Days of oxygen supplementatio*n* (no mechanical ventilation)	2 (2–2.2)	2 (2–2.2)	2 (1–2)	2 (2–2)	NS

## Discussion

In this study, 96% of patients had mild and moderate symptoms. These results are in line with most pediatric reports, showing mainly asymptomatic cases or mild to moderate disease. Regarding the classification of clinical severity, although we follow the one suggested by Dong et al., we consider that it would be necessary to reevaluate its application in clinical practice, as suggested by Buonsenso et al. (6) in their letter to the author. In our center, tomographic images were not performed on patients without clinical manifestations, so the parameter of pathological findings on chest tomography in asymptomatic patients was not used to classify patients as moderate (4–8).

There were no differences in the proportion of male and female patients (53% of patients were male) and the clinical outcomes were not associated with sex. This differs from other reports in adults that showed that clinical infection is more likely to be more severe with higher mortality rates in men (8). It is important to highlight that it has been previously reported in a study from Latin American where girls had a lower frequency of hospitalization and disease severity than boys. Nevertheless, this report included not only COVID-19 patients but also COVID-19 related Multisystem Inflammatory Syndrome (MIS-C) patients, showing limitations in this report due to diverse clinical courses among both pathologies (4–14).

Several studies have shown that the median age of COVID-19 in pediatrics was between 7 and 11 years. In particular, a study in Argentina showed a median age of 7 years and infants under 12 months of age accounted for around 15% of the cases. Interestingly, our study shows a median age of 3 years and 62% of our cohort is under 5 years of age. It is according to data of a multinational experience from Latin America, where the median age of children with COVID-19 was 3 years (IQR 0.6–9 years) (2, 4–7, 11, 15, 16).

Common symptoms at onset of illness were fever and respiratory manifestations, associated with moderate and severe cases; followed by gastrointestinal manifestations (such as vomiting or diarrhea) present mainly in mild disease. It is worth noting that several reports show 45–65% of pediatric cases present fever (14–17). On the other hand, in a study from Latin America, 42/1,010 children had a diagnosis of acute abdomen based on clinical manifestations and 34/38 (89.7%) had a definitive diagnosis of acute apendicitis (18).

Different studies show that comorbidities may be risk factors for poor outcomes with severe infection and hospitalization. Our results align with these observations, showing that 21% of cases had underlying comorbidities and these cases were associated with moderate and severe cases. These comorbidities mainly included chronic pulmonary diseases such as asthma, bronchopulmonary dysplasia and recurrent wheezing. Interestingly, obesity and diabetes mellitus explain the predominant comorbidities in a multicentric study in the United States and onco-hematological diseases were predominant in another study from Argentina (2, 7, 12, 13).

Our results show that 20% of patients required hospital admission and patients with moderate or severe disease had a more prolonged length of stay (LOS). No association between age and hospitalization requirement was found in our study. Similar results were found in previous studies, where hospitalization frequency varied between 6 and 25% reaching 75% depending on the facility setting (14–19).

It is interesting to note that 6% of patients received antimicrobial treatment, mainly in moderate to severe cases due to possible bacterial co-infection. Another study in Latin America showed that 25% of patients received antimicrobial treatment and this was associated with severe cases of COVID-19 and MIS-C (20).

Regarding SARS-CoV-2 viral load, our study showed that higher amounts of viral RNA were found in samples of moderate to severe patients and in patients under 12 months. Several studies carried out in pediatric patients show a significant association between viral load and age, sex, comorbidities, severity of disease and hospitalization requirement. Differences were found in different studies addressing viral load and age. In particular, one study in Chile showed higher viral load in children under 10 years, in another study in New York this was associated with patients under 1 year, and finally in Chicago it was reported for children under 5 years (21–31).

Importantly, in our study no patient received a specific treatment, required admission to PICU nor required mechanical ventilation. No deaths were reported. These results are in line with reports from other healthcare settings, where critical cases and mortality are rarely observed. In Argentina, according to published data from the National Ministry of Health, two age groups of pediatric patients required PICU admission more frequently and showed higher mortality rates, children under 12 months and children between 10 and 14 years. It is important to note, that mortality in Argentina represents 0.5% of pediatric cases and was strongly associated with comorbidities (2). Despite the fact that our hospital covers a large part of Quilmes city population, it has no PICU facilities. Nevertheless, there were no COVID-19 pediatric cases involved in this study that required to be transferred to a PICU of another hospital. This shows that our analyzed population presented mainly mild to moderate disease with low frequency of severe cases and no critical disease.

Our study has several limitations. Firstly, due to the retrospective design, the loss of information in the clinical records is possible. The data collection has been carried out rigorously by the researchers, with a detailed record trying to reduce the risk of bias that this design represents. Secondly, due to the characteristics of the hospital in which it was carried out, it is possible that the population included is not completely representative of the pediatric cases in our country. However, since transfer to other more complex hospitals was not necessary, we can affirm that the patients included represent the best possible sample of the population served. It is important to note that one of the critical limitations of this study lies in considering the cycle threshold (CT) value as an indicator of viral load. In this regard, several variables might affect CT values such as collection technique, specimen type, sampling time, transport and storage conditions as well as nucleic acid extraction, viral RNA load, real time PCR efficiency and CT value determination method. Some of these limitations are thoroughly discussed by several authors and some modifications are proposed to reduce evaluation errors, such as normalization of CT values (32, 33). In spite of its limitations, correlations between SARS-CoV-2 Ct values and patient-related outcomes still have a clinical utility in the context the pandemic in a General Hospital in Latin America. Lastly, other important limitation is that we have no critical cases.

## Conclusion

Our study shows that the viral load of SARS-CoV-2 in respiratory samples, determined indirectly and inversely by the cycle threshold, was significantly correlated with moderate to severe cases and with age, since infants younger than 12 months showed higher amounts of RNA. Disease severity was associated with more frequent and longer hospitalization.

## Data Availability Statement

The raw data supporting the conclusions of this article will be made available by the authors, without undue reservation.

## Ethics Statement

The studies involving human participants were reviewed and approved by Hospital Zonal General de Agudos Dr. Isidoro G. Iriarte. Written informed consent from the participants' legal guardian/next of kin was not required to participate in this study in accordance with the national legislation and the institutional requirements.

## Author Contributions

MB designed the study. MB, SG, GC, MZ, LS, AC, and HF carried out and analyzed the data statistically and revised the manuscript. MB, SG, AC, and GC collected the information. All authors read and approved the final manuscript.

## Conflict of Interest

The authors declare that the research was conducted in the absence of any commercial or financial relationships that could be construed as a potential conflict of interest.

## Publisher's Note

All claims expressed in this article are solely those of the authors and do not necessarily represent those of their affiliated organizations, or those of the publisher, the editors and the reviewers. Any product that may be evaluated in this article, or claim that may be made by its manufacturer, is not guaranteed or endorsed by the publisher.
